# Lipids, biomarkers, and subclinical atherosclerosis in treatment-naive HIV patients starting or not starting antiretroviral therapy: Comparison with a healthy control group in a 2-year prospective study

**DOI:** 10.1371/journal.pone.0237739

**Published:** 2020-08-20

**Authors:** Silvana Di Yacovo, Maria Saumoy, José Luís Sánchez-Quesada, Antonio Navarro, Dmitri Sviridov, Manuel Javaloyas, Ramon Vila, Anton Vernet, Hann Low, Judith Peñafiel, Benito García, Jordi Ordoñez-Llanos, Daniel Podzamczer

**Affiliations:** 1 HIV and STD Unit, Infectious Disease Service, Hospital Universitari de Bellvitge, Bellvitge Biomedical Research Institute, Hospitalet de Llobregat, Spain; 2 Biomedical Research Institute IIB Sant Pau, Barcelona, Spain, Biochemistry and Molecular Biology Department, Universitat Autònoma, Barcelona, Spain; 3 Baker Heart and Diabetes Institute, Melbourne, VIC, Australia; 4 Internal Medicine Service, Hospital de Viladecans, Viladecans, Spain; 5 Vascular Surgery Service, Hospital Universitari de Bellvitge, Hospitalet de Llobregat, Spain; 6 Department of Mechanical Engineering, Universitat Rovira i Virgili, Tarragona, Spain; 7 Biostatistics Unit, Hospital Universitari de Bellvitge, Hospitalet de Llobregat, Spain; 8 University of Barcelona, Barcelona, Spain; University of Texas Medical Branch at Galveston, UNITED STATES

## Abstract

**Objective:**

To assess the effect of HIV infection and combined antiretroviral therapy (c-ART) on various proatherogenic biomarkers and lipids and to investigate their relationship with subclinical atherosclerosis in a cohort of treatment-naive HIV-infected patients.

**Methods:**

We performed a prospective, comparative, multicenter study of 2 groups of treatment-naive HIV-infected patients (group A, CD4>500 cells/μL, not starting c-ART; and group B, CD4<500 cells/μL, starting c-ART at baseline) and a healthy control group. Laboratory analyses and carotid ultrasound were performed at baseline and at months 12 and 24. The parameters measured were low-density lipoprotein (LDL) particle phenotype, lipoprotein-associated phospholipase A2 (Lp-PLA2), interleukin-6 (IL-6), high-sensitivity C-reactive protein (hs-CRP), sCD14, sCD163, monocyte chemoattractant protein-1(MCP-1), and asymmetric dimethylarginine (ADMA). A linear mixed model based on patient clusters was used to assess differences in biomarkers between the study groups and over time.

**Results:**

The study population comprised 62 HIV-infected patients (group A, n = 31; group B, n = 31) and 22 controls. Age was 37 (30–43) years, and 81% were men. At baseline, the HIV-infected patients had a worse LDL particle phenotype and higher plasma concentration of sCD14, sCD163, hs-CRP, and LDL-Lp-PLA2 than the controls. At month 12, there was an increase in total cholesterol (p = 0.002), HDL-c (p = 0.003), and Apo A-I (p = 0.049) and a decrease in sCD14 (p = <0.001) and sCD163 (p<0.001), although only in group B. LDL particle size increased in group B at month 24 (p = 0.038). No changes were observed in group A or in the healthy controls. Common carotid intima-media thickness increased in HIV-infected patients at month 24 (Group A p = 0.053; group B p = 0.048). Plasma levels of sCD14, sCD163, and hs-CRP correlated with lipid values.

**Conclusions:**

In treatment-naive HIV-infected patients, initiation of c-ART was associated with an improvement in LDL particle phenotype and inflammatory/immune biomarkers, reaching values similar to those of the controls. HIV infection was associated with progression of carotid intima-media thickness.

## Introduction

Persons with HIV (PWH) have a higher cardiovascular risk than non–HIV-infected individuals [1]. In addition to traditional cardiovascular risk factors, which are more frequent in the PWH, and the deleterious effect of some antiretroviral drugs on lipid and glucose metabolism, HIV infection itself seems to contribute greatly to overall cardiovascular risk [2]. HIV infection may be associated with atherogenesis through at least 2 mechanisms: chronic inflammation and immune activation that are not completely reversed by combined antiretroviral therapy (c-ART) and disturbances in lipoprotein metabolism.

Compared with healthy individuals, PWH who are not receiving c-ART have higher plasma levels of the inflammatory, coagulation, and immune activation biomarkers that are related to cardiovascular disease in the general population. Viral suppression achieved with c-ART is associated with normalization of various biomarkers, although others remain persistently high even when c-ART is started at an early stage of infection [3–7]. In PWH, some of these biomarkers are associated with increased subclinical carotid and coronary artery atherosclerosis [8–10], as well as cardiovascular events and all-cause mortality [11, 12].

Lipid disorders, which are common in PWH, can be related to the infection itself or to specific antiretroviral drugs [13, 14]. An atherogenic lipoprotein phenotype has been described in treatment-naïve PWH and is characterized by low levels of high-density lipoprotein cholesterol (HDL-c), high triglyceride concentrations, and a predominance of small dense LDL (sd-LDL) particles [15, 16]. LDL particles can be separated into subfractions that differ in physicochemical properties, such as size, charge, density, and lipid/protein composition and thus lead to atherogenicity. Sd-LDL particles have been strongly associated with coronary artery disease in the general population and in PWH [17, 18]. Several studies in PWH have assessed the phenotype of LDL particles in various scenarios [19–24]. However, to our knowledge, only one has assessed the relationship between oxidized LDL particles and markers of inflammation and immune activation in treatment-naïve HIV-infected patients commencing c-ART [25].

The aims of this study were to investigate the effect of HIV infection and c-ART on LDL particle phenotype and atherogenesis-related biomarkers in a cohort of treatment-naïve HIV-infected patients and to assess the relationship between these variables and subclinical carotid atherosclerosis. The data we report can be added to findings published recently by our group on other atherogenesis-related pathways, mainly metabolism of HDL [26]. To our knowledge, no previous studies have compared biomarkers of cardiovascular disease in treatment-naïve HIV-infected patients starting or not starting c-ART and a healthy control group.

## Materials and methods

### Study design, patient population, and endpoints

We performed a prospective, observational, multicenter study of treatment-naive HIV-infected patients recruited in 2 metropolitan hospitals in Spain (Hospital Universitari de Bellvitge and Hospital de Viladecans, Barcelona) and a hospital in Australia (Baker Heart and Diabetes Institute, Melbourne, VIC). HIV-infected patients were divided into 2 groups based on the need to begin c-ART in accordance with European guidelines at the time the study was designed [27]: group A, with a CD4 cell count >500 cells/μL, who did not start c-ART; and group B, with a CD4 count <500 cells/μL, who started c-ART at the baseline visit. To be included, patients had to be aged ≥18 years with HIV infection and have never received c-ART. The exclusion criteria were diabetes mellitus, previous cardiovascular disease, secondary dyslipidemia, malignant disease, any active infection or inflammatory disease, body mass index (BMI) >30 kg/m^2^, and pregnancy. The control group consisted of healthy, HIV-negative, healthcare workers. Group A patients whose CD4 count had decreased to <500 cells/μL were recommended to start c-ART and were not included in the follow-up analyses.

The local ethics committees approved the protocol, and written informed consent was obtained from all participants prior to their inclusion in the study.

We recorded and compared changes in LDL particle phenotype and inflammatory biomarker levels in the 3 groups over a 2-year follow-up period. We investigated the association between infection-related variables (HIV viral load and CD4 cell count) and LDL particle phenotype and inflammatory biomarkers and examined correlations between these variables and subclinical atherosclerosis evaluated using carotid ultrasound.

### Clinical assessment

At baseline and months 12 and 24, patients underwent a clinical assessment, laboratory analyses, and carotid ultrasound. At each visit, smoking, c-ART use, and other medications (lipid-lowering, antidiabetic, and antihypertensive agents) were recorded. The estimated overall cardiovascular risk was calculated using the Framingham score. Blood pressure and anthropometric data (height, weight, and waist and hip circumferences) were recorded.

### Laboratory methods

A venous blood sample was collected (after an overnight fast), centrifuged at 4°C, and stored at −80ºC until analysis. All laboratory measurements were performed at a central laboratory in Barcelona (Biochemistry Department, IIB, Sant Pau Hospital). Samples were shipped to the central laboratory at –80ºC, and aliquots were thawed only once before the analysis; the biomarkers analyzed have been shown to remain stable when preserved at this temperature [28]. Total cholesterol (TC), triglycerides, and glucose were measured using standard enzymatic methods: immunoturbidometry for apolipoprotein B (Apo B) and apolipoprotein A-I (Apo A-I) and a homogeneous direct method for HDL-c. All assays were from Roche Diagnostics (Basel, Switzerland). LDL-c was calculated using the Friedewald formula when the triglyceride concentration was <3.4 mmol/L or was measured after separation of lipoproteins by ultracentrifugation at 105,000 × g for 18 hours at 48ºC when the triglyceride level was >3.4 mmol/L. The central laboratory ensures internal and external quality control programs for the usual lipid parameters.

LDL particle phenotype was determined using gel electrophoresis in 3% polyacrylamide tubes with a commercially available system (Lipoprint, Quantimetrix Co., Redondo Beach, CA, USA). This method can separate 9 LDL subfractions: LDL1 to LDL3, which are large buoyant LDL particles (lb-LDL), and LDL4 to LDL9, which are sd-LDL particles. The results are expressed as LDL particle size (Å), percentage of sd-LDL particles, and cholesterol content in sd-LDL and lb-LDL particles.

We followed the method of Sánchez-Quesada et al [29] to determine lipoprotein-associated phospholipase A2 (Lp-PLA2) activity in serum (total Lp-PLA2) and in lipoproteins using 2-thio-PAF as the substrate (Cayman Chemical Company, Ann Arbor, MI, USA). We also assessed its distribution in LDL and HDL particles. Methods for the measurement of these non-standard lipid variables such as LDL phenotyping or measurement of Lp-PLA2 have been validated in previous publications [22, 23, 29]

High-sensitivity C-reactive protein (hs-CRP) was quantified using an immunocolorimetry assay (Roche Diagnostics, Basel, Switzerland) in a Cobas c501/6000 autoanalyzer. The remaining biomarkers were measured using an enzyme-linked immunosorbent assay, as follows: interleukin-6 (IL-6, eBioscience), monocyte chemoattractant protein-1 (MCP-1, eBioscience), soluble CD14 (sCD14, Hycult Biotech), sCD163 (R&D Systems, Inc.), and asymmetric dimethylarginine (ADMA) (BioVendor). Intra- and inter-assay variabilities were as stated by the manufacturers.

### Carotid ultrasound

High-resolution B-mode ultrasound imaging of the carotid arteries was performed using a Siemens Antares Ultrasound system with a 5- to 10-MHz linear array probe. The carotid bifurcation was screened for plaque starting at 3 cm proximal to the flow divider and covering up to 1 cm distal to the flow divider (internal carotid). Special care was taken at each follow-up examination to ensure that image of the bifurcation was the same as that obtained in the baseline study. Carotid intima-media thickness (c-IMT) at the far wall of the left and right distal common carotid was measured centrally by the same investigator using the ArtWAS software application, which enabled semiautomatic measurement of 1 cm of the distal common c-IMT [30]. The mean of the 2 sides was used for the analysis. A common c-IMT value exceeding the 75^th^ percentile of a reference population was considered indicative of increased cardiovascular risk [31].

### Statistical analysis

The current is a descriptive and exploratory study in which the biological variability of the analyzed variables was unknown a priori and sample size could not be estimated in advance. Categorical variables were presented as the number of cases and percentages. Continuous variables were presented as mean and standard deviation (SD) or median and interquartile range (IQR), depending on whether data distribution. Normality of variables was assessed with graphs (QQ-Plot, density and standard deviations) and non-normal distributed were log-transformed.

The Pearson correlation coefficient (r) and 95% confidence interval (CI) were calculated to estimate the strength of the linear association between variables at baseline.

Linear mixed model with patient cluster were used to assess differences on LDL particle phenotype and atherogenesis-related biomarkers between study groups and over time. Interaction between study group and time was also assessed. The adjusted mean and 95% confidence interval (CI) were reported and presented in graphics. All conditions of application were assessed.

All analyses were performed with a two-sided significance level of 0.05 and conducted with the use of R software version 3.6.1.

## Results

### Participant characteristics

The study population comprised 84 participants, who were included between June 2012 and December 2013 (62 treatment-nave HIV-infected patients [group A, n = 31 and group B, n = 31] and 22 controls [control group]). Nine participants in group A were from the Baker Heart and Diabetes Institute.

**Fig 1:**
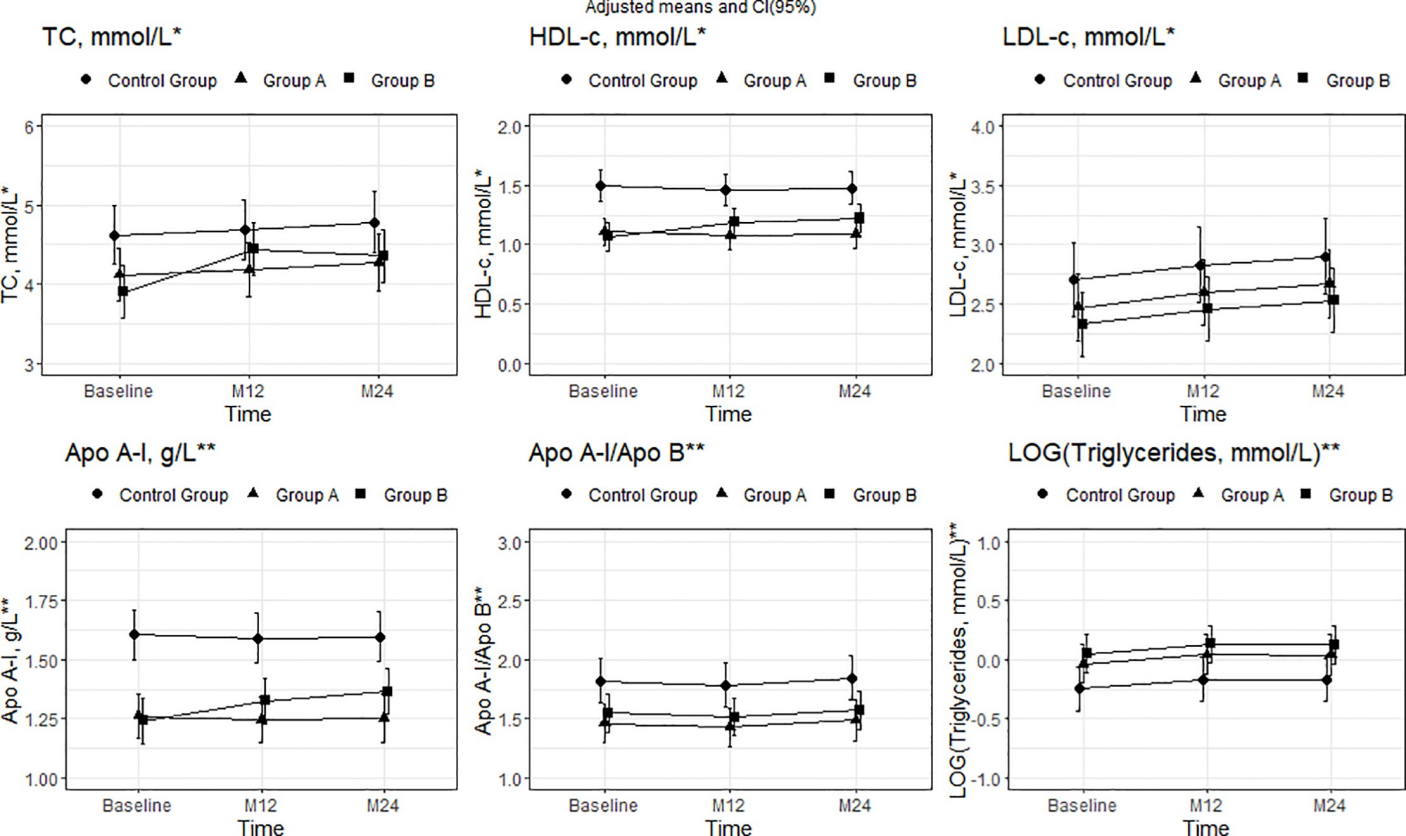
Mean adjusted for group, time, and interaction between group and time and 95% CI in lipid parameters from baseline to months 12 and 24. *Mean adjusted for group, time, and interaction between group B and time. **Mean adjusted for group and time. In TC, the interaction between time and group was statistically significant for group B at 12 months (p = 0.002). In HDL-c and APO A-1, the interaction was statistically significant for group B at 12 months (p = 0.003 and 0.049, respectively) and 24 months (p = 0.001 and 0.008, respectively). Moreover, for all 3 lipid parameters, differences at baseline were statistically significant for group A compared with the control group (p = 0.03, <0.001, and <0.001, respectively) and group B compared with the control group (p = 0.004, <0.001, and <0.001, respectively). In the case of LDL-c and Apo A-I/Apo B, differences at baseline were statistically significant in group B compared with the control group (p = 0.019 and 0.021); baseline differences in group A compared with the control group were statistically significant only for Apo A-I/Apo B (p = 0.017). Abbreviations: TC, total cholesterol; HDL-c, high-density lipoprotein cholesterol.

**Fig 2:**
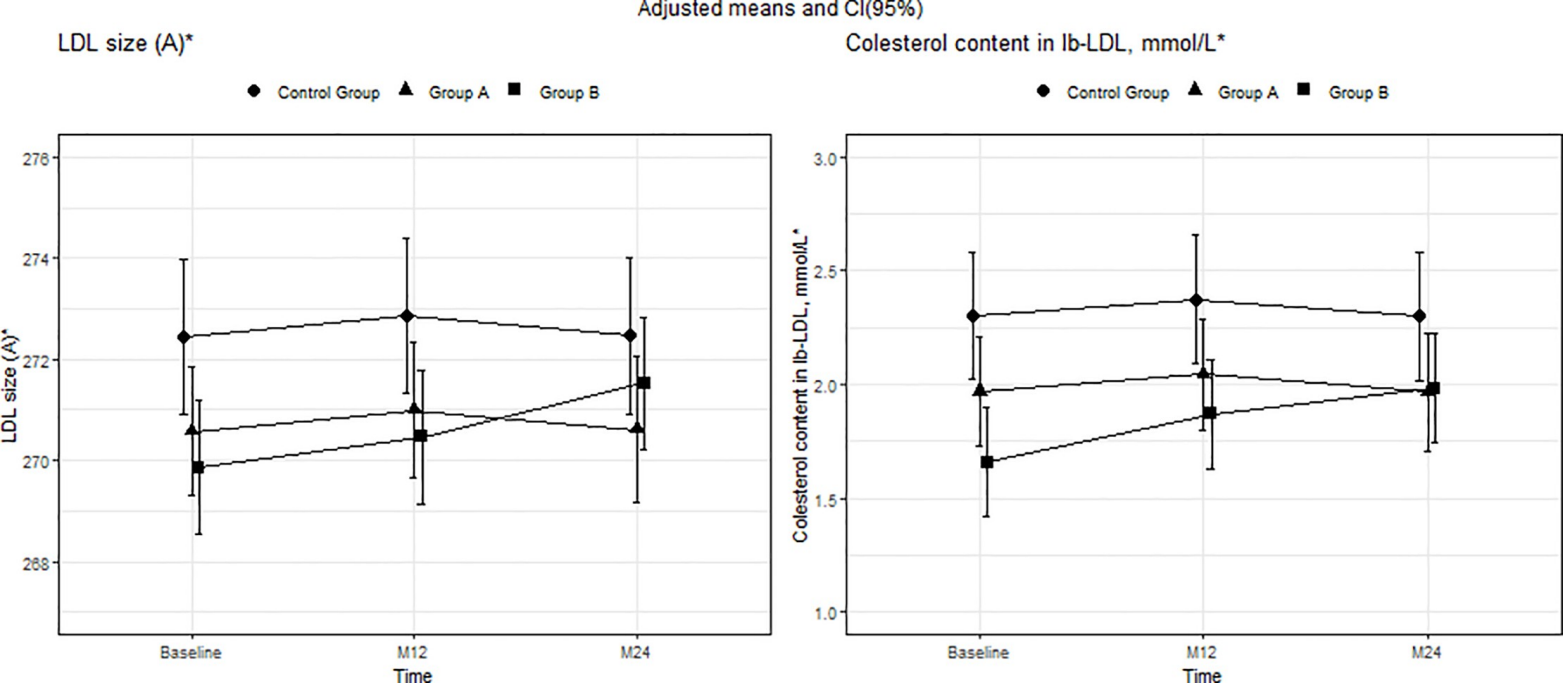
Mean adjusted for group, time, and interaction between group and time and 95% CI in LDL particle phenotype parameters from baseline to months 12 and 24. *Mean adjusted for group, time, and interaction between group B and Time. In LDL size and cholesterol content in lb-LDL particles, the interaction between time and group was statistically significant for group B at 24 months (p = 0.038 and 0.009, respectively). Moreover, in both variables, the differences at baseline were statistically significant for group B compared with the control group (p = 0.011 and 0.001, respectively); baseline differences in group A compared with the control group were statistically significant only for LDL size (p = 0.049). Abbreviations: LDL, low-density lipoprotein cholesterol; lb-LDL, large buoyant LDL particles.

**Fig 3:**
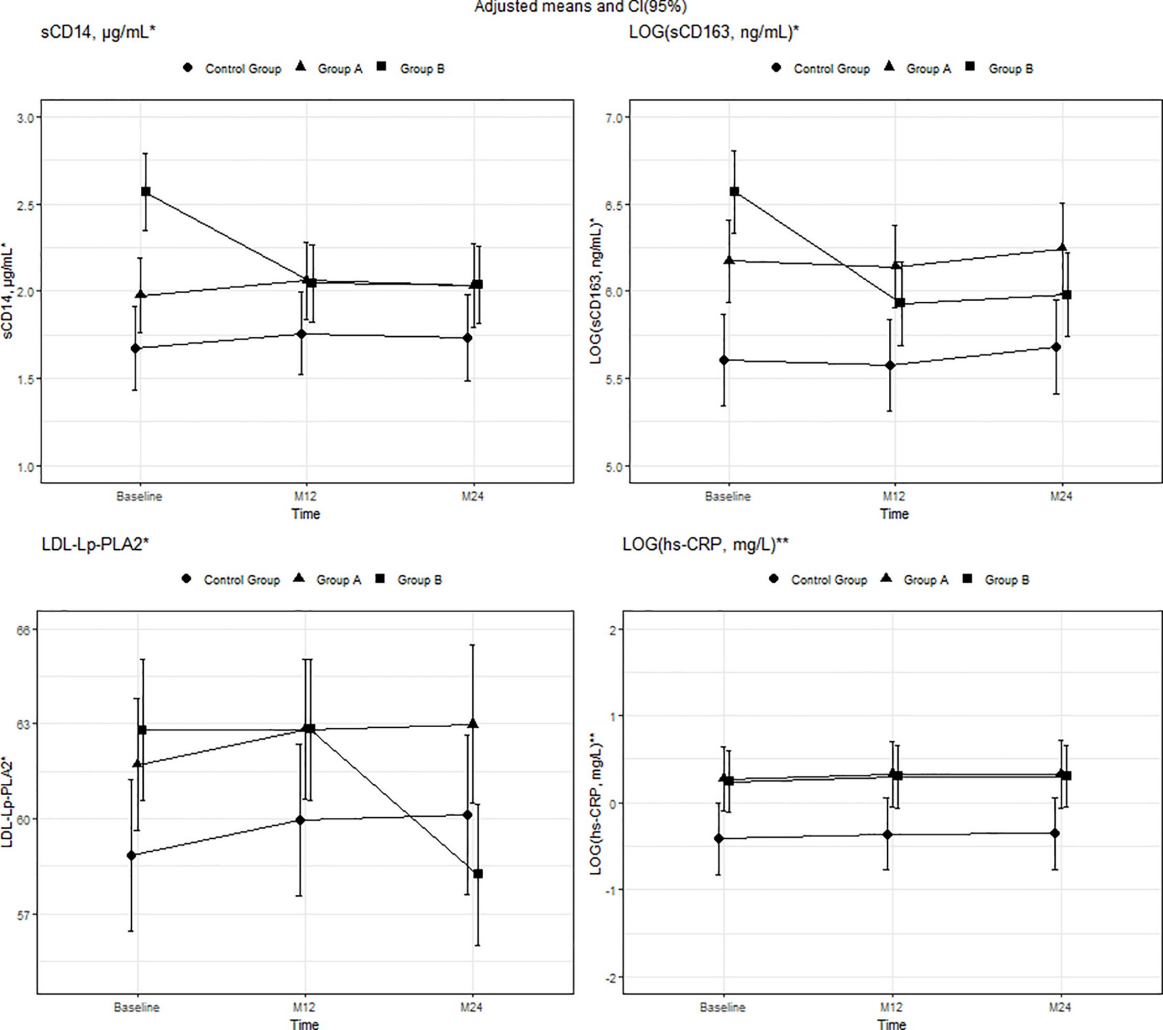
Mean adjusted for group, time, and interaction between group and time and 95% CI in plasma biomarkers from baseline to months 12 and 24. *Mean adjusted for group, time, and interaction between Group B and time. **Mean adjusted for group and time. In sCD14 and LOG(sCD163), the interaction between group and time was statistically significant for group B at 12 months (p<0.001 and <0.001, respectively) and 24 months (p<0.001 and <0.001, respectively). Moreover, differences at baseline were statistically significant in LOG(sCD163) and sCD14 for group A compared with the control group (p = 0.001 and p = 0.038 respectively) and group B compared with the control group (p<0.001 and p<0.001 respectively). In LDL-Lp-PLA2, the interaction between time and group was statistically significant for group B at 24 months (p<0.001); differences at baseline were statistically significant for groups A and B compared with the control group (p = 0.048 and p = 0.01, respectively). In LOG(hs-CRP), the differences at baseline were statistically significant for groups A and B compared with the control group (p = 0.007 and p = 0.002, respectively). Abbreviations: hs-CRP, high-sensitivity C-reactive protein; LDL-Lp-PLA2, lipoprotein-associated phospholipase A2.

The baseline characteristics of the participants are shown in Tables 1 and 2. Mean (SD) age was 37 (8.54) years, and 79.8% were men, with no differences between the groups. Significant differences were found in several lipid and biomarker plasma levels between HIV-infected patients and the control group. Groups A and B had lower plasma levels of TC, HDL-c, Apo A-I, and Apo A-I/Apo B, in addition to lower LDL particle size than the control group. Plasma levels of sCD14, LOG(sCD163), LOG(hs-PCR), and LDL-Lp-PLA2 were higher in both group A and group B than in the control group (Figs 1–3) (Tables 1 and 2).

**Table 1:** Baseline characteristics of the participants.

	All participants N = 83	Control group n = 22	Group A n = 30	Group B n = 31	N
Age, y	37.0 (8.54)	37.4 (8.91)	36.3 (9.06)	37.3 (7.99)	81
Male sex, N (%)	67 (79.8%)	15 (68.2%)	26 (83.9%)	26 (83.9%)	84
Time with HIV infection, months*	21.9 [6.70;50.3]	-	23.1 [6.88;54.8]	19.5 [7.28;44.3]	53
HIV viral load, copies/mL*	20367 [4804;79858]	-	9719 [3656;39416]	47210 [6912;129057]	58
CD4 count, cells/μL	563 (260)	-	734 (193)	415 (216)	58
**CD4%**			31 (26–36)	19 (16–25)	58
**CD4/CD8 ratio**			0.75 (0.47–0.95)	0.32 (0.25–0.48)	58
BMI, kg/m^2^	24.4 (4.28)	23.6 (3.21)	24.6 (5.64)	24.5 (3.10)	75
Waist-to-hip ratio	0.93 (0.07)	0.90 (0.07)	0.91 (0.06)	0.96 (0.07)	65
SBP, mmHg	126 (17.2)	123 (24.5)	122 (11.0)	132 (17.3)	75
DBP, mmHg,	76.8 (10.3)	73.2 (13.3)	75.4 (8.68)	79.9 (9.61)	75
**Cardiovascular risk factors**					
Current smoker, N (%)	28 (35.0%)	6 (31.6%)	12 (40.0%)	10 (32.3%)	80
Hypertension, N (%)	3 (3.75%)	2 (10.5%)	0 (0.00%)	1 (3.23%)	80
Family history of premature CHD, N (%)	15 (18.8%)	5 (26.3%)	5 (16.7%)	5 (16.1%)	80
Framingham risk*	3.90 [1.90;6.70]	2.55 [1.20;5.52]	3.90 [2.10;5.60]	5.15 [2.50;7.90]	71
**Carotid ultrasound**					
Common c-IMT, mm*	0.540 [0.473;0.636]	0.552 [0.517;0.607]	0.539 [0.501;0.649]	0.520 [0.440;0.627]	75
Common c-IMT >75^**th**^ percentile, N (%)	12 (16.2%)	4 (22.2%)	6 (21.4%)	2 (7.14%)	74
Carotid plaque, N (%)	5 (6.49%)	1 (4.76%)	1 (3.70%)	3 (10.3%)	

Abbreviations: BMI, body mass index; c-IMT, carotid intima-media thickness; CHD, coronary heart disease; DBP, diastolic blood pressure; SBP, systolic blood pressure.

*Continuous variables expressed as median [interquartile range]; all other variables are expressed as mean (standard deviation) or N (%).

**Table 2:** Baseline laboratory characteristics of participants.

	All participants N = 83	Control group n = 22	Group A n = 30	Group B n = 31	N
**Lipid variables**					
TC, mmol/L	4.11 [3.55;4.77]	4.78 [4.08;5.23]	3.97 [3.45;4.54]	3.95 [3.33;4.44]	80
HDL-c, mmol/L	1.17 [0.89;1.41]	1.38 [1.29;1.72]	1.07 [0.86;1.39]	1.05 [0.80;1.30]	80
LDL-c, mmol/L	2.40 [1.96;3.04]	2.92 [2.24;3.32]	2.37 [1.95;2.92]	2.37 [1.86;2.60]	80
TC/HDL-c	3.49 [3.00;4.14]	3.03 [2.77;3.67]	3.77 [3.23;4.55]	3.62 [3.19;4.17]	80
Triglycerides, mmol/L	0.88 [0.72;1.12]	0.82 [0.64;1.06]	0.86 [0.72;1.34]	0.95 [0.81;1.18]	80
Apo B, g/L	0.89 [0.74;1.03]	0.94 [0.75;1.06]	0.84 [0.77;0.96]	0.89 [0.70;1.00]	80
Apo A-I, g/L	1.35 [1.14;1.51]	1.58 [1.35;1.70]	1.23 [1.13;1.41]	1.26 [1.06;1.39]	80
Apo A-I/Apo B,	1.48 [1.31;1.76]	1.74 [1.36;2.21]	1.47 [1.30;1.65]	1.44 [1.26;1.68]	80
**LDL particle phenotype**					
LDL size, Å	272 [269;274]	273 [272;274]	272 [270;274]	270 [268;273]	81
sd-LDL particles,%,	18.4 [13.4;31.9]	15.0 [12.2;18.3]	17.9 [13.1;24.1]	29.2 [16.6;34.6]	78
Cholesterol in sd-LDL, mmol/L,	0.49 [0.29;0.74]	0.36 [0.30;0.57]	0.49 [0.24;0.64]	0.61 [0.34;0.82]	78
Cholesterol in lb-LDL, mmol/L,	1.80 [1.46;2.33]	2.39 [1.64;2.78]	1.93 [1.48;2.29]	1.64 [1.30;1.96]	78
**Biomarkers**					
Total Lp-PLA2 activity, μmol/min·mL	18.8 [16.1;21.3]	17.9 [15.2;20.2]	18.9 [16.7;20.9]	19.3 [16.7;22.2]	83
LDL-Lp-PLA2 (%),	62.1 [57.6;66.1]	57.6 [54.6;62.6]	62.6 [59.8;66.0]	62.2 [59.2;67.5]	83
sCD14, μg/mL	1.97 [1.66;2.31]	1.70 [1.54;1.95]	1.86 [1.66;2.13]	2.36 [2.10;3.10]	79
sCD163, ng/mL	478 [284;904]	260 [185;453]	500 [334;710]	836 [423;1195]	80
ADMA, μmol/L	0.52 [0.44;0.58]	0.52 [0.45;0.60]	0.45 [0.38;0.53]	0.54 [0.51;0.57]	77
IL-6, pg/mL	1.13 [0.89;1.36]	0.93 [0.73;1.30]	1.16 [0.98;1.44]	1.13 [0.93;1.32]	80
hs-CRP, mg/L	0.90 [0.50;1.85]	0.45 [0.30;0.80]	1.25 [0.78;2.47]	1.25 [0.60;3.43]	80
MCP-1, pg/mL	77.8 [60.9;89.9]	74.8 [51.0;86.4]	78.9 [62.1;92.2]	77.9 [66.1;89.9]	78

Abbreviations: ADMA, asymmetric dimethylarginine; Apo, apolipoprotein; HDL-c, high density lipoprotein-cholesterol; hs-CRP, high-sensitivity C-reactive protein; IL-6, interleukin-6; lb-LDL, large buoyant low-density lipoprotein; LDL-c, low-density lipoprotein cholesterol; Lp-PLA2, lipoprotein-associated phospholipase A2; MCP-1, monocyte chemoattractant protein-1; sd-LDL, small dense low-density lipoprotein; TC, total cholesterol. Continuous variables are expressed as median [interquartile range].

Group B started c-ART at the beginning of the study. The combined regimen was chosen by the attending physician according to local practice. All patients started 2 nucleoside reverse transcriptase inhibitors plus a protease inhibitor (n = 17), an integrase inhibitor (n = 11), a CCR5 inhibitor (n = 2), or a non-nucleoside reverse transcriptase inhibitor (n = 1).

The CD4 cell count increased a median of 174 (62–346) cells/μL during the study in group B, whereas no change was observed in group A. Viral load was undetectable at months 12 and 24 in 93.3% and 96.6% of participants in group B, respectively.

Because of decreases in CD4 cell count to <500 cells/μL, 3 patients in group A started c-ART during the first year and were not included in the follow-up analyses, and 8 started during the second year and were only included in the month 12 follow-up analysis. In addition, 6 patients in group A (3 during the first year and 3 during the second year), 1 in group B, and 2 controls were lost to follow-up. Therefore, group A included 25 and 14 patients at months 12 and 24 of follow-up, respectively.

### Lipid and LDL particle phenotype changes

Lipid changes were observed only in group B. There was a significant increase in TC, HDL-c, and Apo A-I at month 12. Total cholesterol remained stable at 24 months, and similar values were observed between study groups. HDL-c and Apo A-I had increased further at 24 months. The adjusted means [95% CI] of TC in group B (as indicated by the model) at baseline and 12 and 24 months were 3.9 [3.63;4.16], 4.44 [4.02;4.86], and 4.36 [3.97;4.74], respectively. The adjusted means [95% CI] were 1.07 [0.95;1.18], 1.19 [1.09;1.29], and 1.22 [1.11;1.34] for HDL and 1.24 [1.17;1.32], 1.33 [1.25;1.40] and 1.36 [1.26;1.45] for Apo A-I. No changes were observed in group B for LDL-c, Apo B, TC/HDL-c, the Apo A-I/Apo B ratio, or triglycerides. There were no changes in any of the lipid parameters in group A or in the control group (Figs 1, 2 and 3) (S1 Table of S1 Data).

LDL particle size and cholesterol content in lb-LDL particles improved in group B at month 24. According to the model, the adjusted means [95%CI] at baseline and 12 and 24 months in group B were 269.86 [268.4;271.31], 270.46 [268.71;272.21], and 271.53 [270.54;272.51], respectively, for LDL particle size and 1.66 [1.45;1.87], 1.87 [1.61;2.13], and 1.98 [1.71;2.25] for cholesterol content in lb-LDL. There were no changes in group A or in the control group. At month 24, there were no significant differences in LDL phenotype between the 3 study groups (Fig 2) (S2 Table of S1 Data).

We carried out a correlation analysis with lipid variables including all participants. At baseline, LDL particle size correlated with several lipid variables and BMI (r[95% CI] = –0.261 [–0.463; –0.033]) (Table 2). No correlations were found between lipid variables and HIV viral load or CD4 cell count.

### Changes inflammatory biomarkers

Changes were observed only in group B, with a significant decrease in sCD14 and in LOG(sCD163) at 12 months and a further decrease at 24 months. In line with the model, the adjusted means [95% CI] in group B at baseline and 12 and 24 months were 2.57 [2.27;2.87], 2.05 [1.85;2.25], and 2.03 [1.81;2.26] for sCD14 plasma levels and 6.57 [6.32;6.81], 5.93 [5.66;6.19], and 5.98 [5.77;6.18] for LOG(sCD163). During the study period, no changes were recorded for hs-CRP, which remained higher in group B at month 24 (Fig 3). No changes in IL-6, ADMA, or MCP-1 were observed in any group at 12 and 24 months. Similar values of IL-6, ADMA, and MCP-1 between study groups were expected at 12 and 24 months (S2 Table of S1 Data).

Total Lp-PLA2 remained largely unchanged in all 3 groups during the study, although a significant decrease in LDL-Lp-PLA2 was observed at 24 months. Consistent with the model, the adjusted means [95% CI] for LDL-Lp-PLA2 in group B at baseline and 12 and 24 months were 62.79 [60.59;64.99], 62.82 [60.63;65.00], and 58.24 [55.41;61.07] (Fig 3) (S2 Table of S1 Data).

A correlation analysis based on CD4 cell count, HIV viral load, and the lipid variables was carried out at baseline. sCD14, sCD163, and hs-CRP levels correlated with those of several lipid variables (TC, HDL-c, LDL-c, Apo A-I, TC/HDL-c, Apo A-I/Apo B, and triglycerides). Various correlations were also found between plasma biomarkers (Table 3).

**Table 3:** Correlations between plasma biomarkers, lipids, and HIV status at baseline.

	LDL size	Total Lp-PLA2	IL-6	sCD14	LOG(sCD163)	LOG(hs-CRP)
**LOG(HIV viral load)**	0.02 [-0.24;0.28]	-0.05 [-0.3;0.22]	0.09 [-0.18;0.35]	-0.04 [-0.31;0.23]	0.2 [-0.07;0.44]	0.23 [-0.04;0.47]
**CD4 count**	0.12 [-0.14;0.37]	-0.05 [-0.31;0.21]	0.31 [0.05;0.53]	-0.02 [-0.29;0.26]	-0.24 [-0.48;0.03]	-0.13 [-0.39;0.14]
**LOG(CD4/CD8)**	0.2 [-0.06;0.44]	-0.24 [-0.47;0.02]	0.29 [0.02;0.52]	-0.02 [-0.29;0.25]	-0.26 [-0.49;0.01]	-0.15 [-0.4;0.13]
**TC**	-0.01 [-0.23;0.22]	0.11 [-0.11;0.32]	-0.1 [-0.32;0.12]	-0.02 [-0.25;0.21]	-0.31 [-0.5;-0.09]	-0.24 [-0.44;-0.02]
**HDL-c**	0.43 [0.22;0.59]	-0.34 [-0.52;-0.13]	-0.17 [-0.38;0.06]	-0.1 [-0.32;0.13]	-0.3 [-0.49;-0.09]	-0.3 [-0.49;-0.09]
**LDL-c**	0.13 [-0.09;0.34]	0.25 [0.03;0.44]	-0.13 [-0.34;0.1]	0.01 [-0.21;0.24]	-0.29 [-0.48;-0.08]	-0.22 [-0.42;0]
**TC/HDL-c**	-0.54 [-0.68;-0.36]	0.47 [0.27;0.62]	0.08 [-0.15;0.29]	0.07 [-0.16;0.29]	0.11 [-0.11;0.32]	0.18 [-0.04;0.38]
**LOG (triglycerides)**	-0.69 [-0.79;-0.55]	0.16 [-0.06;0.37]	0.13 [-0.1;0.34]	0.05 [-0.18;0.27]	0.12 [-0.1;0.33]	0.19 [-0.03;0.39]
**Apo B**	-0.19 [-0.39;0.04]	0.3 [0.09;0.49]	-0.1 [-0.31;0.13]	0.04 [-0.19;0.26]	-0.22 [-0.42;0]	-0.17 [-0.37;0.06]
**Apo A-I**	0.21 [-0.01;0.41]	-0.17 [-0.37;0.05]	-0.08 [-0.29;0.15]	-0.2 [-0.41;0.03]	-0.34 [-0.52;-0.13]	-0.28 [-0.47;-0.07]
**Apo A-I/Apo B**	0.29 [0.07;0.48]	-0.42 [-0.59;-0.22]	0.04 [-0.18;0.26]	-0.13 [-0.34;0.1]	-0.09 [-0.3;0.13]	-0.09 [-0.3;0.14]
**LDL size**	–	-0.19 [-0.39;0.03]	-0.04 [-0.27;0.18]	-0.14 [-0.35;0.09]	-0.09 [-0.31;0.13]	-0.24 [-0.44;-0.02]
**Total Lp-PLA2**	–	–	0 [-0.22;0.23]	-0.03 [-0.26;0.19]	-0.05 [-0.27;0.18]	0.03 [-0.19;0.24]
**IL-6**	–	–	–	0.01 [-0.22;0.24]	0.28 [0.06;0.47]	0.2 [-0.02;0.41]
**sCD14**	–	–	–	–	–	0.48 [0.29;0.63]
**LOG(sCD163)**	–	–	–	–	–	0.09[-0.14;0.32]

Abbreviations: Apo, apolipoprotein; HDL-c, high-density lipoprotein cholesterol; hs-CRP, high-sensitivity C-reactive protein; IL-6, interleukin-6; LDL-c, low-density lipoprotein cholesterol; Lp-PLA2, lipoprotein-associated phospholipase A2; MCP-1, monocyte chemoattractant protein-1; sd-LDL, small dense low-density lipoprotein; TC, total cholesterol. No significant correlations were observed for MCP-1 or asymmetric dimethylarginine. Therefore, they are not shown in the table.

### Changes in carotid intima-media thickness, anthropometric measures, and cardiovascular risk score

**Fig 4:**
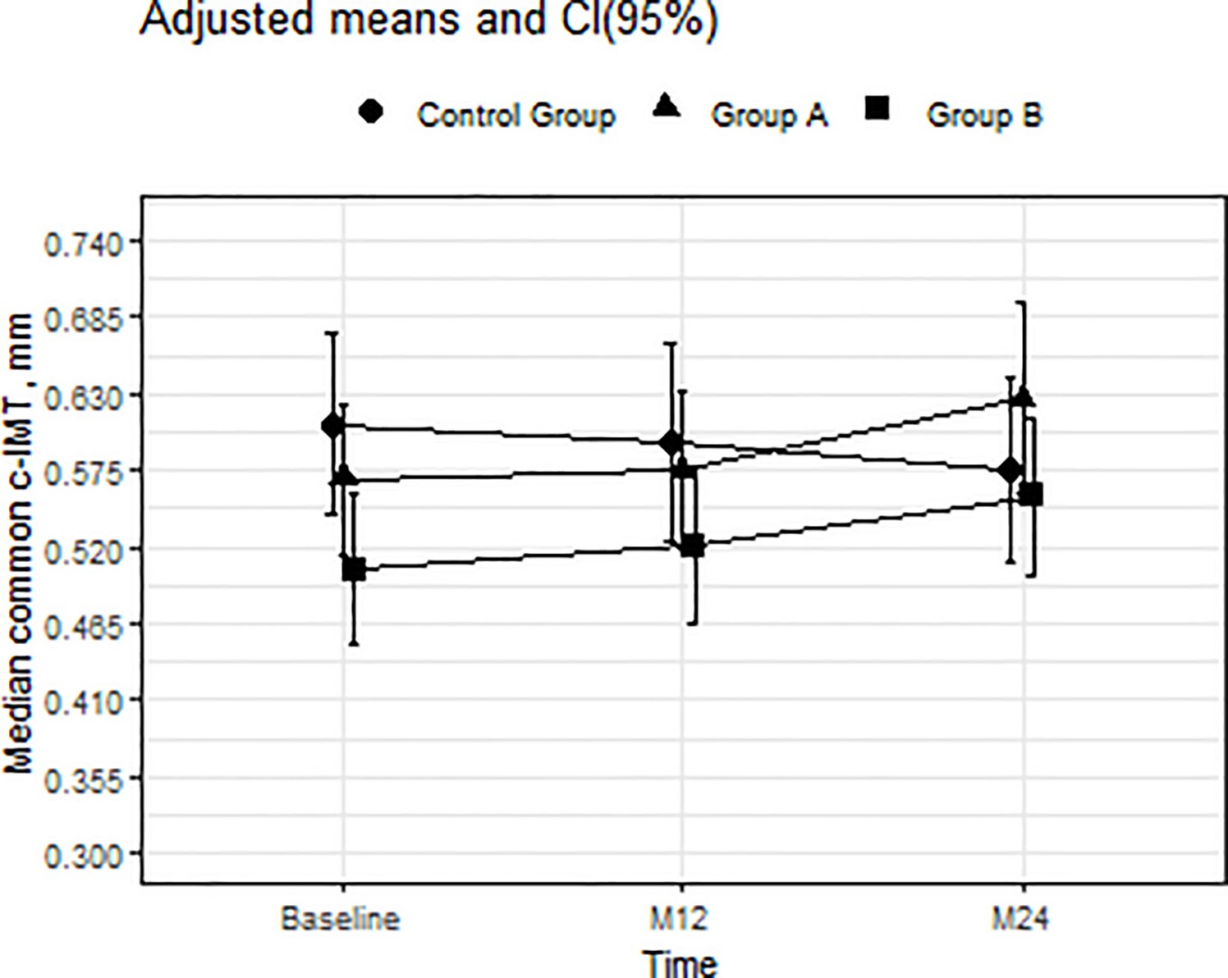
Mean adjusted for group, time, and interaction between group and time and 95% CI in common c-IMT from baseline to months 12 and 24. The interaction between group and time was statistically significant at 24 months for group B (p = 0.048) and almost significant for group A (p = 0.053). The interaction was statistically significant for group B compared with the control group (p = 0.015). Abbreviations: c-IMT, carotid intima-media thickness.

At baseline, common c-IMT was thicker in the control group than in group B (Table 1 and Fig 4). There was a significant increase in common c-IMT in group B at 24 months (p = 0.048); the difference was almost significant in group A (p = 0.53). The adjusted means [95%CI] for c-IMT in group B were 0.504 [0.45;0.558], 0.522 [0.448;0.586], and 0.557 [0.501;0.614]. No changes were recorded in the percentage of participants with common c-IMT over the 75^th^ percentile.

At baseline, common c-IMT correlated with age (r[95% CI], 0.387 [0.174;0.565]), Framingham score (Log) (r[95% CI], 0.362 [0.133;0.554]), TC (r[95% CI], 0.257 [0.029;0.46]), LDL-c (r[95% CI], 0.289 [0.063;0.487]), and BMI (r[95% CI], 0.285 [0.052;0.489]), but not with biomarkers, HIV viral load, or CD4.

Cardiovascular risk measured by Framingham score (LOG) at baseline was similar in all 3 study groups and did not change significantly during the study period.

A sensitivity analysis revealed a non statistically significant interaction between patients with a regimen containing a protease inhibitor or not (S3 Table of S1 Data).

## Discussion

In this study, we assessed biological and ultrasound biomarkers of atherogenesis in treatment-naive HIV-infected patients who initiated or did not initiate c-ART and in a healthy control group over a 2-year follow-up period. The study was designed before the recommendation that all PWH should start c-ART regardless of CD4 cell count. Therefore, we had a unique opportunity to assess a series of atherogenesis-related variables in a group of treatment-naive HIV-infected patients and compare the results with those of patients starting c-ART. Our main finding was that levels of inflammatory and immune activation markers were higher and the lipid profile more unfavorable in treatment-naive patients than in controls. In addition, starting c-ART was associated with decreased levels of some biomarkers and an improvement in LDL particle phenotype and some lipids. All of these biomarkers reached levels similar to those of the controls, with the exception of hs-CRP, which remained higher. Finally, c-ART could not prevent an increase in common c-IMT, although the percentage of participants with subclinical atherosclerosis remained low.

A pro-atherogenic lipid pattern mediated by cytokine dysregulation in chronic inflammation and infection has previously been described in naive HIV-infected patients [13–16]. Consistent with these findings and compared with the controls, the treatment-naive patients in our study had lower HDL-c levels, a tendency toward a higher TC/HDL-c ratio, and a more unfavorable LDL particle phenotype (lower LDL particle size and percentage of lb-LDL particles). sd-LDL particles are more atherogenic owing to various mechanisms: they are more avidly taken up by macrophages and are more susceptible to oxidative modification, they have a greater propensity for transport into the arterial subendothelial space, and they have greater potential for binding to arterial wall proteoglycans than lb-LDL particles [17].

Our findings point to a relationship between a proatherogenic lipid pattern and immune activation and inflammation. At baseline, the highest sCD163, sCD14, and hs-CRP biomarker levels were associated with the lowest TC and HDL-c levels and the highest TC/HDL and triglyceride levels. Recent reviews of the relationship between lipid homeostasis, inflammation, and immune activation related to microbial translocation showed that, on the one hand, the consequences of microbial translocation (such as increased lipopolysaccharide level) can lower expression of ATP-binding cassette transporters, thus reducing reverse cholesterol transport from the arterial wall to HDL particles. On the other hand, immune activation can also enhance the activity of reactive oxygen species and enzymes such as Lp-PLA2, thereby rendering LDL and HDL particles more pro-inflammatory [32, 33]. Data on the association between immune activation, inflammation, and lipids in PWH are scarce [10, 25, 34, 35]. sCD14, sCD163, and hs-CRP have been associated with oxidized forms of LDL and HDL in treatment-naive patients and after initiation of c-ART [25].

In our study, initiation of c-ART was associated with an increase in plasma lipid concentrations to levels similar to those of the controls, which can be considered a return to a “healthy” state [25]. LDL particle phenotype also improved in patients starting c-ART, despite the non-significant increase in triglycerides, a key determinant of LDL particle size and density. Consistent with the results of the present study, our group previously reported an improvement in LDL particle phenotype with initiation of ritonavir-boosted darunavir, whereas phenotype worsened in patients receiving ritonavir-boosted atazanavir, in association with a greater increase in triglyceride levels [23]. In contrast, in a study assessing the characteristics of LDL particles in treatment-naïve patients starting atazanavir/ritonavir, darunavir/ritonavir, or raltegravir plus tenofovir-emtricitabine, Kelesidis et al [24] found a decrease in LDL particle numbers only with raltegravir and no changes in LDL particle size in any treatment arm. The laboratory methods used by Kelesidis et al differed from ours and may thus explain the contrasting findings. In summary, HIV infection is related to a pro-atherogenic LDL particle phenotype that improves after initiation of c-ART, reaching values similar to those of controls despite increases in the levels of traditional lipid parameters, which may be associated with the improvement in immune activation observed in our study.

We observed a beneficial effect of suppressive c-ART on biomarkers of inflammation (LDL-Lp-PLA2) and immune activation (sCD14 and sCD163), which reached concentrations similar to those of the controls. Nonetheless, some degree of inflammation persisted, as evidenced by the continuously elevated levels of hs-CRP. Previous studies have also shown improvements in biomarkers after suppressive c-ART, although continuously high plasma concentrations of some biomarkers of inflammation (hs-CRP, IL-6, TNF) and immune activation (sCD14) have been reported by several authors [3–6, 36]. Recovery of CD4 cell counts and, to a lesser extent, control of HIV viral load have been associated with a decrease in some biomarkers of inflammation and immune activation [5]. It has been amply demonstrated that c-ART alone cannot normalize the plasma levels of all biomarkers. Other factors, such as intestinal microbial translocation, persistent viral replication, and co-infections (eg, by hepatitis virus and cytomegalovirus), which are not reverted by c-ART, are thought to contribute to persistence of inflammation [3, 5, 7].

Carotid-IMT and plaque are predictors of future cardiovascular events in the general population and have been used as surrogate markers of cardiovascular disease [37]. At baseline in our study, c-IMT was higher in the healthy control group and was associated with traditional cardiovascular risk factors (age and Framingham score) but not with HIV-specific factors. While common c-IMT has been assessed in naive HIV-infected patients in various studies [38–42], only 2 included a healthy control group, and no differences between HIV or healthy groups were found [39,42]. Hileman et al [39] reported a similar increase in common c-IMT in both treatment-naïve HIV-infected patients without c-ART and healthy controls followed over a period of 2 years; moreover, this increase was independently associated with hs-CRP. In contrast, we recorded a greater increase in common c-IMT in HIV-infected patients who started or did not start c-ART, but not in the control group, suggesting that HIV infection itself plays a role that may be mediated by hs-CRP, which remained higher in HIV-infected patients. It is noteworthy that the percentage of participants with c-IMT over the 75^th^ percentile, which was associated with increased cardiovascular risk, did not change during the study.

Our study has several major limitations. The small sample size at baseline and the withdrawal of some participants in group A (mainly because of initiation of c-ART due to disease progression and, less frequently, because of loss to follow-up) decrease the power of the study and may introduce bias in the results. Moreover, variability in the repeated measures of c-IMT could hamper interpretation of the results. Therefore, findings for c-IMT must be interpreted with caution, mainly in group A. Nonetheless, the fact that significant changes were recorded confers validity to the study. The prospective design and inclusion of treatment-naïve HIV-infected patients and healthy controls provided the opportunity to study the effect of HIV infection and c-ART on various biomarkers and on subclinical atherosclerosis. The use of different types of c-ART with specific effects on lipids, biomarkers, and even c-IMT [4, 24] may have confounded the results; however, a sensitivity analysis among patients receiving and not receiving protease inhibitors did not reveal differences in the variables analyzed. At baseline there were several differences in the variables analyzed between the control group and the two HIV-infected groups, related mainly with the own HIV-infection. The population included was young and predominantly male, thus precluding generalization of the results to older patients and women.

## Conclusions

In conclusion, this study shows that c-ART normalizes the lipid profile and improves LDL particle phenotype and immune activation, although some degree of inflammation persists. However, c-ART was unable to prevent progression of common c-IMT. As c-ART alone did not fully normalize the values of biomarkers of inflammation, additional strategies are needed to reduce the non–AIDS-defining disorders associated with chronic inflammation.

## Supporting information

S1 Data (RTF)
