# Supplementary material for: Lipids, biomarkers, and subclinical atherosclerosis in treatment-naive HIV patients starting or not starting antiretroviral therapy: Comparison with a healthy control group in a 2-year prospective study
Source: PLoS One. 2020 Aug 20;15(8):e0237739. doi: 10.1371/journal.pone.0237739 (PMC7446923; doi:10.1371/journal.pone.0237739)
Supplement: S1 Data — (RTF) [file pone.0237739.s001.rtf]

	TC, mmol/L	HDL-c, mmol/L	LDL-c, mmol/L	LOG(TC/HDL-c)	
Predictors	Estimates	CI	p	Estimates	CI	p	Estimates	CI	p	Estimates	CI	p	
Intercept	4.62	4.25 – 5.00	<0.001	1.50	1.37 – 1.63	<0.001	2.70	2.39 – 3.01	<0.001	1.15	1.04 – 1.27	<0.001	
Group A vs Control Group	-0.51	-0.98 – -0.03	0.037	-0.39	-0.56 – -0.22	<0.001	-0.23	-0.64 – 0.17	0.263	0.20	0.05 – 0.34	0.009	
Group B vs Control Group	-0.72	-1.22 – -0.22	0.005	-0.43	-0.61 – -0.25	<0.001	-0.37	-0.77 – 0.03	0.067	0.14	-0.00 – 0.28	0.058	
12 months vs baseline	0.06	-0.13 – 0.26	0.535	-0.03	-0.09 – 0.03	0.305	0.13	0.02 – 0.24	0.026	0.03	-0.02 – 0.08	0.260	
24 months vs baseline	0.16	-0.06 – 0.38	0.157	-0.02	-0.09 – 0.05	0.602	0.20	0.08 – 0.32	0.001	0.02	-0.04 – 0.07	0.576	
Group B vs 12 months	0.48	0.17 – 0.79	0.003	0.15	0.05 – 0.25	0.002							
Group B vs 24 months	0.30	-0.03 – 0.62	0.075	0.17	0.07 – 0.28	0.001							
Random Effects					
ó2	0.23	0.02	0.13	0.02	
ô00	0.64 npac	0.08 npac	0.48 npac	0.06 npac	
ICC	0.73	0.79	0.79	0.71	
N	81 npac	81 npac	81 npac	81 npac	
Observations	221	221	221	221	
Marginal R2 / Conditional R2	0.079 / 0.756	0.207 / 0.830	0.047 / 0.801	0.069 / 0.730	
AIC	502.221	28.031	389.054	4.654	
S1 Table. Linear mixed model for lípid variables between group A, group B and control group. 

 	Apo B	Apo A-I	Apo A-I/Apo B	LOG(Triglycerides, mmol/L)	
Predictors	Estimates	CI	p	Estimates	CI	p	Estimates	CI	p	Estimates	CI	p	
Intercept	0.91	0.82 – 0.99	<0.001	1.60	1.50 – 1.71	<0.001	1.82	1.64 – 2.00	<0.001	-0.25	-0.43 – -0.07	0.007	
Group A vs Control Group	-0.03	-0.14 – 0.08	0.609	-0.34	-0.48 – -0.21	<0.001	-0.36	-0.60 – -0.12	0.003	0.21	-0.02 – 0.44	0.076	
Group B vs Control Group	-0.04	-0.15 – 0.07	0.518	-0.36	-0.50 – -0.22	<0.001	-0.27	-0.50 – -0.04	0.022	0.30	0.07 – 0.52	0.010	
12 months vs baseline	0.04	0.01 – 0.08	0.012	-0.01	-0.07 – 0.04	0.631	-0.03	-0.11 – 0.04	0.400	0.08	-0.01 – 0.17	0.074	
24 months vs baseline	0.03	-0.00 – 0.07	0.080	-0.01	-0.07 – 0.06	0.835	0.03	-0.06 – 0.11	0.554	0.07	-0.02 – 0.17	0.120	
Group B vs 12 months				0.10	0.00 – 0.19	0.040							
Group B vs 24 months				0.13	0.03 – 0.23	0.009							
Random effects					
ó2	0.01	0.02	0.06	0.08	
ô00	0.04 npac	0.05 npac	0.16 npac	0.14 npac	
ICC	0.76	0.70	0.73	0.65	
N	81 npac	81 npac	81 npac	81 npac	
Observations	221	220	220	221	
Marginal R2 / Conditional R2	0.013 / 0.758	0.240 / 0.771	0.091 / 0.752	0.070 / 0.671	
AIC	-135.183	-24.752	200.366	235.140	


S2 Table. Linear mixed model for LDL particle phenotype and biomarkers between group A, group B and control group.
 	LDL size	Cholesterol in sd-LDL, mmol/L	Cholesterol in lb-LDL	Lp-PLA2 activity-total	
Predictors	Estimates	CI	p	Estimates	CI	p	Estimates	CI	p	Estimates	CI	p	
Intercept	272.44	270.93 – 273.95	<0.001	0.50	0.35 – 0.66	<0.001	2.30	2.03 – 2.58	<0.001	18.37	16.76 – 19.99	<0.001	
Group A vs Control Group	-1.86	-3.71 – -0.01	0.049	0.06	-0.14 – 0.26	0.539	-0.33	-0.68 – 0.02	0.061	0.29	-1.79 – 2.37	0.783	
Group B vs Control Group	-2.58	-4.57 – -0.58	0.011	0.07	-0.12 – 0.27	0.457	-0.64	-1.01 – -0.28	0.001	0.79	-1.26 – 2.84	0.451	
12 months vs baseline	0.42	-0.53 – 1.36	0.386	-0.00	-0.09 – 0.08	0.979	0.07	-0.08 – 0.22	0.346	0.18	-0.49 – 0.85	0.606	
24 months vs baseline	0.03	-1.02 – 1.08	0.949	0.08	-0.01 – 0.17	0.096	-0.00	-0.17 – 0.16	0.970	-0.22	-0.93 – 0.50	0.550	
Group B vs 12 months	0.19	-1.28 – 1.66	0.800				0.14	-0.10 – 0.38	0.244				
Group B vs 24 months	1.63	0.09 – 3.18	0.038				0.33	0.08 – 0.57	0.009				
Random effects	
ó2	5.18	0.07	0.13	4.54	
ô00	8.50 npac	0.09 npac	0.31 npac	12.57 npac	
ICC	0.62	0.58	0.71	0.73	
N	81 npac	78 npac	78 npac	84 npac	
Observations	220	215	216	226	
Marginal R2 / Conditional R2	0.066 / 0.646	0.013 / 0.581	0.099 / 0.734	0.008 / 0.737	
AIC	1128.303	184.657	361.320	1166.222	

 	LDL-Lp-PLA2	sCD14, µg/mL	LOG(sCD163, ng/mL)	ADMA, µmol/L	
Predictors	Estimates	CI	p	Estimates	CI	p	Estimates	CI	p	Estimates	CI	p	
Intercept	58.84	56.47 – 61.21	<0.001	1.67	1.44 – 1.91	<0.001	5.60	5.34 – 5.86	<0.001	0.55	0.33 – 0.77	<0.001	
Group A vs Control Group	2.86	0.02 – 5.70	0.048	0.30	0.02 – 0.59	0.038	0.57	0.24 – 0.90	0.001	-0.05	-0.33 – 0.23	0.737	
Group B vs Control Group	3.95	0.70 – 7.19	0.017	0.89	0.57 – 1.22	<0.001	0.97	0.61 – 1.32	<0.001	0.14	-0.13 – 0.41	0.309	
12 months vs baseline	1.12	-0.75 – 2.99	0.240	0.08	-0.09 – 0.26	0.358	-0.03	-0.19 – 0.13	0.695	-0.02	-0.16 – 0.11	0.726	
24 months vs baseline	1.27	-0.85 – 3.39	0.241	0.06	-0.14 – 0.26	0.574	0.07	-0.11 – 0.25	0.424	-0.05	-0.20 – 0.09	0.463	
Group B vs 12 months	-1.09	-4.07 – 1.89	0.474	-0.60	-0.89 – -0.32	<0.001	-0.61	-0.86 – -0.35	<0.001				
Group B vs 24 months	-5.82	-8.96 – -2.68	<0.001	-0.59	-0.89 – -0.29	<0.001	-0.66	-0.93 – -0.40	<0.001				
Random effects					
ó2	21.69	0.19	0.16	0.19	
ô00	17.75 npac	0.19 npac	0.28 npac	0.16 npac	
ICC	0.45	0.49	0.64	0.46	
N	84 npac	81 npac	81 npac	78 npac	
Observations	225	220	221	215	
Marginal R2 / Conditional R2	0.076 / 0.492	0.151 / 0.567	0.183 / 0.709	0.020 / 0.474	
AIC	1425.038	395.952	389.835	369.067	

 	IL-6, pg/mL	LOG(hs-CRP, mg/L)	MCP-1, <U+03C1>g/mL	
Predictors	Estimates	CI	p	Estimates	CI	p	Estimates	CI	p	
Intercept	1.03	0.85 – 1.22	<0.001	-0.41	-0.82 – -0.01	0.045	71.85	62.95 – 80.74	<0.001	
Group A vs Control Group	0.32	0.08 – 0.55	0.007	0.69	0.17 – 1.21	0.010	5.38	-5.83 – 16.59	0.347	
Group B vs Control Group	0.09	-0.13 – 0.32	0.415	0.66	0.15 – 1.16	0.011	4.88	-5.96 – 15.73	0.377	
12 months vs baseline	0.07	-0.03 – 0.17	0.200	0.06	-0.16 – 0.27	0.605	-1.63	-6.68 – 3.42	0.527	
24 months vs baseline	0.04	-0.07 – 0.14	0.503	0.06	-0.17 – 0.29	0.595	1.55	-3.78 – 6.88	0.568	
Group B vs 12 months										
Group B vs 24 months										
Random effects				
ó2	0.10	0.47	239.25	
ô00	0.13 npac	0.69 npac	286.12 npac	
ICC	0.57	0.60	0.54	
N	80 npac	81 npac	78 npac	
Observations	216	221	211	
Marginal R2 / Conditional R2	0.068 / 0.595	0.075 / 0.626	0.013 / 0.550	
AIC	257.536	606.960	1853.998	


S3 Table. Linear mixed model for lípids, LDL particle phenotype and biomarkers between participants taking protease inhibitors and no taking protease inhibitors in group B * 
  	TC, mmol/L 	HDL-c, mmol/L 	LDL-c, mmol/L 	LOG(TC/HDL-c) 	
Predictors 	Estimates 	CI 	p 	Estimates 	CI 	p 	Estimates 	CI 	p 	Estimates 	CI 	p 	
Intercept 	3.55 	3.08 – 4.02 	<0.001 	1.05 	0.89 – 1.20 	<0.001 	1.99 	1.61 – 2.38 	<0.001 	1.27 	1.12 – 1.41 	<0.001 	
IP vs No IP 	0.62 	0.05 – 1.19 	0.034 	0.03 	-0.16 – 0.23 	0.737 	0.48 	-0.01 – 0.97 	0.057 	0.10 	-0.09 – 0.29 	0.300 	
12 months vs baseline 	0.54 	0.24 – 0.84 	0.001 	0.12 	0.04 – 0.20 	0.002 	0.24 	0.05 – 0.42 	0.012 	-0.01 	-0.09 – 0.07 	0.860 	
24 months vs baseline 	0.46 	0.15 – 0.76 	0.003 	0.16 	0.08 – 0.23 	<0.001 	0.29 	0.11 – 0.47 	0.002 	-0.04 	-0.12 – 0.04 	0.305 	
Random effects	
σ2 	0.36 	0.02 	0.13 	0.02 	
τ00 	0.51 npac 	0.07 npac 	0.42 npac 	0.06 npac 	
ICC 	0.59 	0.74 	0.76 	0.71 	
N 	30 npac 	30 npac 	30 npac 	30 npac 	
Observations 	90 	90 	90 	90 	
Marginal R2 / Conditional R2 	0.149 / 0.647 	0.050 / 0.749 	0.116 / 0.789 	0.032 / 0.718 	
AIC 	228.010 	10.595 	160.961 	9.817 	
*Interaction between groups and time was not statistically significant. 
  	Apo B 	Apo A-I 	Apo A-I/Apo B 	LOG(Triglycerides, mmol/L) 	
Predictors 	Estimates 	CI 	p 	Estimates 	CI 	p 	Estimates 	CI 	p 	Estimates 	CI 	p 	
Intercept 	0.79 	0.68 – 0.90 	<0.001 	1.19 	1.08 – 1.30 	<0.001 	1.56 	1.34 – 1.79 	<0.001 	-0.07 	-0.34 – 0.20 	0.611 	
IP vs No IP 	0.12 	-0.02 – 0.26 	0.105 	0.09 	-0.04 – 0.23 	0.181 	-0.09 	-0.38 – 0.20 	0.548 	0.17 	-0.17 – 0.50 	0.329 	
12 months vs baseline 	0.07 	0.01 – 0.13 	0.018 	0.08 	0.01 – 0.15 	0.019 	0.01 	-0.10 – 0.12 	0.914 	0.14 	-0.02 – 0.30 	0.076 	
24 months vs baseline 	0.04 	-0.02 – 0.10 	0.209 	0.12 	0.05 – 0.19 	0.001 	0.09 	-0.02 – 0.20 	0.105 	0.09 	-0.07 – 0.25 	0.278 	
Random Effects	
σ2 	0.01 	0.02 	0.05 	0.10 	
τ00 	0.03 npac 	0.03 npac 	0.14 npac 	0.18 npac 	
ICC 	0.70 	0.60 	0.75 	0.65 	
N 	30 npac 	30 npac 	30 npac 	30 npac 	
Observations 	90 	89 	89 	90 	
Marginal R2 / Conditional R2 	0.083 / 0.729 	0.091 / 0.634 	0.019 / 0.756 	0.036 / 0.665 	
AIC 	-36.253 	-23.185 	72.392 	122.439 	
*Interaction between groups and time was not statistically significant. 


  	LDL size 	Cholesterol in sd-LDL, mmol/L 	Cholesterol in lb-LDL 	Lp-PLA2 activity-total 	
Predictors 	Estimates 	CI 	p 	Estimates 	CI 	p 	Estimates 	CI 	p 	Estimates 	CI 	p 	
Intercept 	270.18 	268.31 – 272.04 	<0.001 	0.52 	0.34 – 0.71 	<0.001 	1.47 	1.14 – 1.80 	<0.001 	18.81 	16.53 – 21.09 	<0.001 	
IP vs No IP 	-0.58 	-2.86 – 1.70 	0.619 	0.14 	-0.08 – 0.36 	0.222 	0.34 	-0.08 – 0.76 	0.110 	1.29 	-1.67 – 4.24 	0.394 	
12 months vs baseline 	0.61 	-0.77 – 1.99 	0.389 	0.02 	-0.11 – 0.16 	0.734 	0.21 	0.03 – 0.39 	0.019 	-0.23 	-1.32 – 0.86 	0.684 	
24 months vs baseline 	1.67 	0.29 – 3.05 	0.018 	-0.02 	-0.15 – 0.12 	0.806 	0.32 	0.15 – 0.50 	<0.001 	-0.89 	-1.98 – 0.21 	0.112 	
Random Effects	
σ2 	7.69 	0.07 	0.12 	4.81 	
τ00 	7.80 npac 	0.07 npac 	0.29 npac 	15.88 npac 	
ICC 	0.50 	0.50 	0.70 	0.77 	
N 	31 npac 	30 npac 	30 npac 	31 npac 	
Observations 	93 	90 	90 	93 	
Marginal R2 / Conditional R2 	0.035 / 0.521 	0.034 / 0.518 	0.102 / 0.731 	0.026 / 0.774 	
AIC 	500.086 	80.391 	148.608 	487.065 	
*Interaction between groups and time was not statistically significant. 


  	Lp-PLA2 activity-LDL-Lp-PLA 	sCD14, µg/mL 	LOG(sCD163, ng/mL) 	ADMA, µmol/L 	
Predictors 	Estimates 	CI 	p 	Estimates 	CI 	p 	Estimates 	CI 	p 	Estimates 	CI 	p 	
Intercept 	61.38 	58.25 – 64.52 	<0.001 	2.39 	2.07 – 2.70 	<0.001 	6.44 	6.12 – 6.77 	<0.001 	0.70 	0.30 – 1.09 	0.001 	
IP vs No IP 	2.56 	-1.32 – 6.44 	0.195 	0.32 	-0.07 – 0.70 	0.105 	0.22 	-0.18 – 0.63 	0.274 	-0.08 	-0.56 – 0.39 	0.729 	
12 months vs baseline 	0.03 	-2.16 – 2.22 	0.978 	-0.52 	-0.74 – -0.30 	<0.001 	-0.64 	-0.85 – -0.44 	<0.001 	0.08 	-0.20 – 0.36 	0.584 	
24 months vs baseline 	-4.55 	-6.74 – -2.36 	<0.001 	-0.53 	-0.76 – -0.31 	<0.001 	-0.59 	-0.80 – -0.39 	<0.001 	-0.04 	-0.32 – 0.24 	0.793 	
Random Effects	
σ2 	19.39 	0.20 	0.16 	0.31 	
τ00 	23.61 npac 	0.22 npac 	0.25 npac 	0.33 npac 	
ICC 	0.55 	0.53 	0.61 	0.52 	
N 	31 npac 	30 npac 	30 npac 	30 npac 	
Observations 	93 	90 	90 	90 	
Marginal R2 / Conditional R2 	0.128 / 0.607 	0.174 / 0.612 	0.190 / 0.681 	0.006 / 0.521 	
AIC 	586.456 	170.215 	162.594 	209.059 	
*Interaction between groups and time was not statistically significant. 
  	IL-6, pg/mL 	LOG(hs-CRP, mg/L) 	MCP-1,g/mL 	
Predictors 	Estimates 	CI 	p 	Estimates 	CI 	p 	Estimates 	CI 	p 	
Intercept 	1.10 	0.93 – 1.27 	<0.001 	0.09 	-0.42 – 0.59 	0.739 	81.12 	69.29 – 92.95 	<0.001 	
IP vs No IP 	0.15 	-0.05 – 0.35 	0.149 	0.50 	-0.12 – 1.11 	0.117 	-4.89 	-18.87 – 9.09 	0.493 	
12 months vs baseline 	0.02 	-0.11 – 0.15 	0.772 	-0.13 	-0.46 – 0.20 	0.449 	-3.67 	-13.22 – 5.88 	0.451 	
24 months vs baseline 	-0.09 	-0.22 – 0.04 	0.160 	-0.12 	-0.46 – 0.21 	0.463 	-1.48 	-11.03 – 8.07 	0.761 	
Random Effects	
σ2 	0.06 	0.43 	340.73 	
τ00 	0.06 npac 	0.59 npac 	256.39 npac 	
ICC 	0.46 	0.58 	0.43 	
N 	30 npac 	30 npac 	30 npac 	
Observations 	90 	90 	87 	
Marginal R2 / Conditional R2 	0.061 / 0.495 	0.059 / 0.602 	0.014 / 0.437 	
AIC 	68.905 	242.856 	776.985 	
*Interaction between groups and time was not statistically significant. 
Abbreviations: Apo, apolipoprotein; HDL-c, high density lipoprotein-cholesterol; LDL-c, low-density lipoprotein cholesterol; TC, total cholesterol.
Abbreviations: ADMA, asymmetric dimethylarginine; hs-CRP, high-sensitivity C-reactive protein; IL-6, interleukin-6; lb-LDL, large buoyant low-density lipoprotein; Lp-PLA2, lipoprotein-associated phospholipase A2; MCP-1, monocyte chemoattractant protein-1; sd-LDL, small dense low-density lipoprotein.
